# Supplementary material for: Variation in the Promoter Region of the MC4R Gene Elucidates the Association of Body Measurement Traits in Hu Sheep
Source: Int J Mol Sci. 2019 Jan 9;20(2):240. doi: 10.3390/ijms20020240 (PMC6358852; doi:10.3390/ijms20020240)
Supplement: Supplementary file 1 [file ijms-20-00240-s001.pdf]

Variation in the promoter region of MC4R gene Elucidate the Association of Body Measurement Traits in Hu sheep

Shishay Girmay[1, 2], Guiqiong Liu[1, 2], Xunping* Jiang[1, 2], Yun Yu[1], Teketay Wassie[2], Dandan Du[2], Huang Jing[2], Liu chenghui[2]

[1] Laboratory of Small Ruminant Genetics, Breeding and Reproduction, Huazhong Agricultural University, Wuhan 430070, People's Republic of China

[2] Key Laboratory of Agricultural Animal Genetics, Breeding and Reproduction of the Ministry of Education Wuhan 430070, People's Republic of China

* Correspondence: liuguiqiong@mail.hzau.edu.cn (G.L.); Tel. +8618007166396:

Received: date; Accepted: date; Published: date

Table S1. The SNPs in the Sheep MC4R that alter the cis-regulatory element/s.

No	Transcription factor	Transcription factor binding sites	Target strand	SNPs
1.	COUP-TF (nuclear receptor)	tag**C**ccttgcaccc	+	-1131C>T
2.	Chop-cEBP (bZIP)	g**C**ccttgcaccc	-	-1131C>T
3.	Activator Protein 1 (AP-1)	a**G**agaaattg	+	-1038 G>A
4.	Activator Protein 1 (AP-1)	aga**G**aaattg	+	-1036G>T
5.	SP-1	attgaga**G**gg	+	-1026G>A
6.	HNF-3beta (Forkhead)	**G**ggtaaataaat	-	-1026G>A
7.	RXR-VDR (Nuclear Receptor)	**G**ggtaaataaattca	+	-1026G>A
8.	C/EBPalp	ttttttt**G**tt	+	-943G>T
9.	HNF-3	tttttt**G**ttt	+	-943G>T
10.	Hb	tt**G**ttttttt	+	-943G>T
11.	Hunchback (Zn-Finger, C2H2)	ttttttttt**G**	_	-943G>T
12.	HFH-2 (Forkhead)	tttttttt**G**ttt	+	-943G>T
13.	HMG-IY (HMG)	tttttttt**G**ttttttt	-	-943G>T
14.	Sox-5 (HMG)	ttt**G**ttt	-	-943G>T
15.	TEF-1 (TEA)	tgcat**G**cctccg	+	-287G>A
16.	SP- 1(zinc finger transcription factor)	t**G**cctccgac	+	-287G>A
17.	C/EB	**G**gatttccaa	+	-206G>A
18.	p65	ta**G**gatttcc	+	-206G>A
19.	SOX17 (HMG)	gccact**G**tc	+	-103G>C

Shown the SNPs in the Sheep MC4R promoter region that changed the Transcription factor binding sites. The capital letters in bold are mutated sequence, which causes a change in the transcription factors. Notably, all of the eight SNPs changes one or more Transcription factor binding sites.

Table S2 Estimated value of linkage equilibrium analysis between eight SNPs in MC4R gene of Hu Sheep population.

	SNP1	SNP2	SNP3	SNP4	SNP5	SNP6	SNP7	SNP8
SNP1		0.8153	0.7599	**0.9996**	0.7143	0.8149	0.847	0.8985
SNP2	0.6886		**0.9524**	0.7969	0.7994	0.8377	0.6762	0.7981
SNP3	0.7146	0.8553		0.8206	0.8364	0.7824	0.7913	0.7886
SNP4	**0.8889**	0.7568	0.776		0.6927	**0.943**	0.8197	**0.9048**
SNP5	0.5861	0.7766	0.7297	0.6391		0.7949	0.542	0.7205
SNP6	0.7856	0.7338	0.7631	**0.8698**	0.6765		**0.9378**	**0.9302**
SNP7	0.6383	0.6034	0.6341	0.6947	0.4978	0.733		**0.902**
SNP8	**0.818**	0.7404	0.7634	**0.8839**	0.6494	**0.8783**	0.7467	

The correlation coefficients D′ and r^2 between eight SNPs are shown in the above and below diagonal of this table, respectively.

Table S3 Haplotypes and haplotype frequencies of eight SNPs in sheep MC4R in the Hu sheep population.

	SNP1	SNP2	SNP3	SNP4	SNP5	SNP6	SNP7	SNP8	Frequency
**H1**	**C**	**G**	**G**	**G**	**G**	**G**	**G**	**C**	**0.6321**
**H2**	**T**	**A**	**T**	**A**	**T**	**A**	**A**	**G**	**0.0847**
**H3**	**T**	**A**	**T**	**A**	**T**	**A**	**G**	**G**	**0.0534**
H4	T	G	G	G	G	G	G	C	0.0339
H5	T	A	T	A	G	A	A	G	0.0278
H6	C	G	T	G	G	G	G	C	0.0196
H7	T	G	G	A	G	A	A	G	0.0127
H8	C	G	G	G	T	G	G	C	0.0125
H9	T	G	G	A	G	A	G	G	0.0124
H10	T	G	T	A	G	A	A	G	0.0122
H11	C	A	T	G	T	G	G	C	0.0102

Haplotypes and haplotype frequencies of eight SNPs in sheep MC4R in the Hu sheep population determined using the SNPStat (https://www.snpstats.net/start.htm) online server. The haplotype frequencies >0.05 are indicated in bold.

Table S4 Primers designed for SNP detection in the Sheep MC4R gene

ID	Primers Sequence(5′-3′)	Nucleotide position*	Annealing temperature (℃)
MC4R-1	F: TGTTGCTGAGCAGCTTCTCTTAC	-2000/-1190	58.7
	R: ACTCACTGACATTGGTGGGC		
MC4R-2	F: AACCTGAGTGTTGCCTAGCTATT	-1346/-401	60
	R: GCTTTGCTTTGAGTGCCAGAA		
MC4R-3	F: CCTCTGAATTGGGGTTGCCT	-559/+88	60
	R: ACTCACTGACATTGGTGGGC		

*The nucleotide positions of primers are shown according to their location relative to the first start codon of the MC4R gene. These three overlapped primers were designed using primer 3Plus (http://www.bioinformatics.nl/cgi-bin/primer3plus/primer3plus.cgi)

Table S5 Primers designed for qRT-PCR

ID	Primers Sequence(5′-3′)	Product length (bp)	Annealing temperature (℃)
MC4R	F: GGCCAGGCTCCACATTAAGA	118	60
	R: GCAGACAACAAAGACCCCGA		
β-actin	F: CCAACCGTGAGAAGATGACC	97	61
	R: CCAGAGGCGTACAGGGACAG		

Table S6 Primers used for cloning the MC4R promoter.

Plasmids	Primer	Sequence 5′to 3′
pGL3-2000	-2000	CCG**GGTACC**TGTTGCTGAGCAGCTTCTCTTAC
pGL3-1489	-1489	CGG**GGTACC**GGAGGTGATGAATATGCTGAGT
pGL3-1207	-1207	CGG**GGTACC**GGAGAAATAGCTGATCTTAGTGCA
pGL3-880	-880	CGG**GGTACC**TGCTGTGCAGCAAAGCAAAT
pGL3-369	-369	CGG**GGTACC**AGCTGATGAGAGCATGCGC
Common antisense	+88	CCG**TCTAGA**ACTCACTGACATTGGTGGGC

*The nucleotide positions of primers are shown according to their location relative to the first start codon of the MC4R gene. These three overlapped primers were designed using primer 3Plus (http://www.bioinformatics.nl/cgi-bin/primer3plus/primer3plus.cgi). Restriction endonuclease sequences are shown in bold.

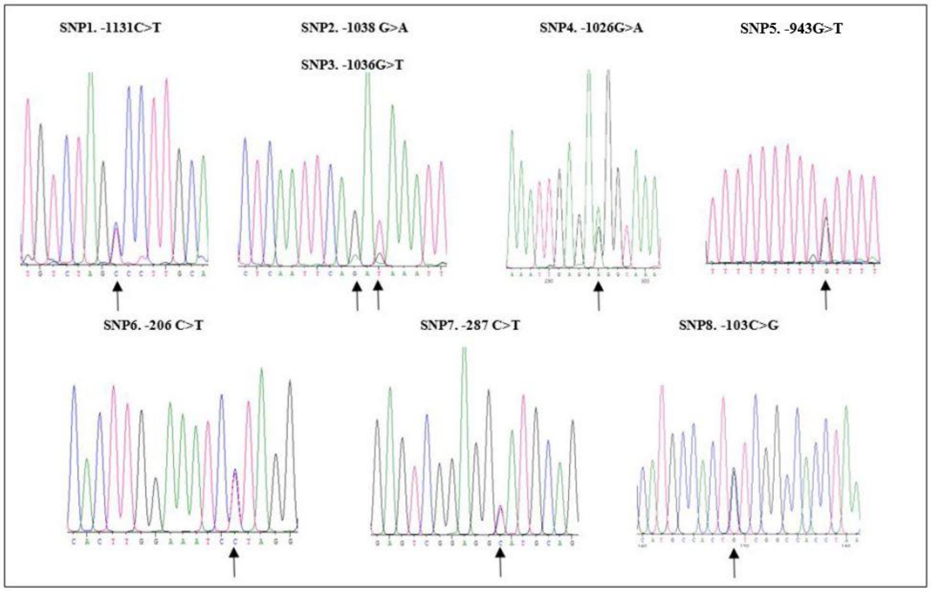

Figure S1. Nucleotide sequences of the MC4R gene showed eight SNPs in the Hu sheep promoter region. The arrow indicates the position of the SNPs. The number and the nucleotide indicate the SNP position relative to the start codon.

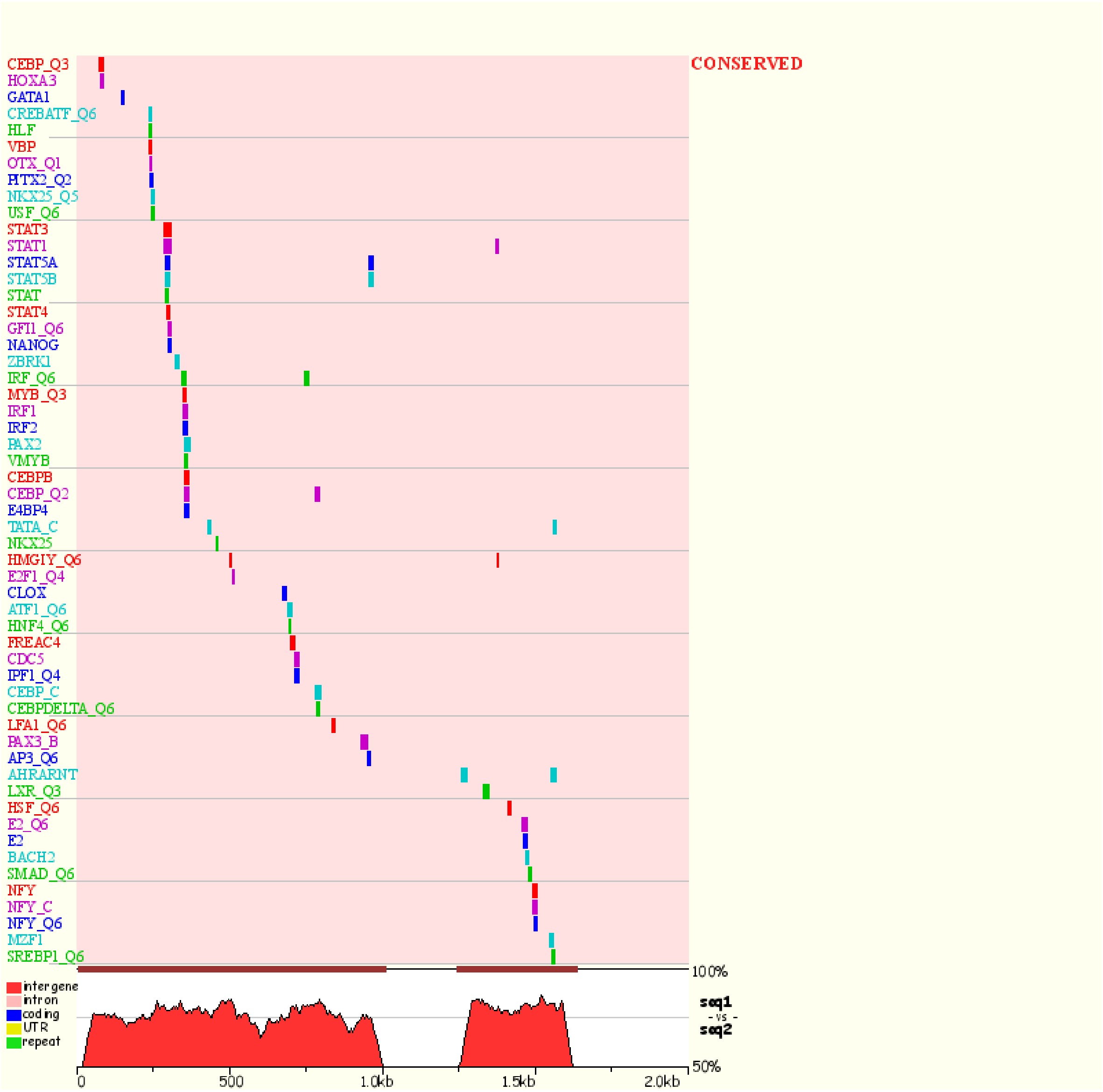

Figure S2. Picture constructed by zPicture for a 2.1-kb potential promoter region from Sheep MC4R gene chromosome 23 (Chr 23: 59,385,576-59,386,574 reverse strand) and an orthologous Pig MC4R (NCBI accession number: NM_214173). The default parameters (>100 bp/>70% ID) were used to highlight intronic (pink) and intragenic (red) conserved elements. Exons are in blue and repeats are in green. Inverted regions shaded in gray.

CONREAL

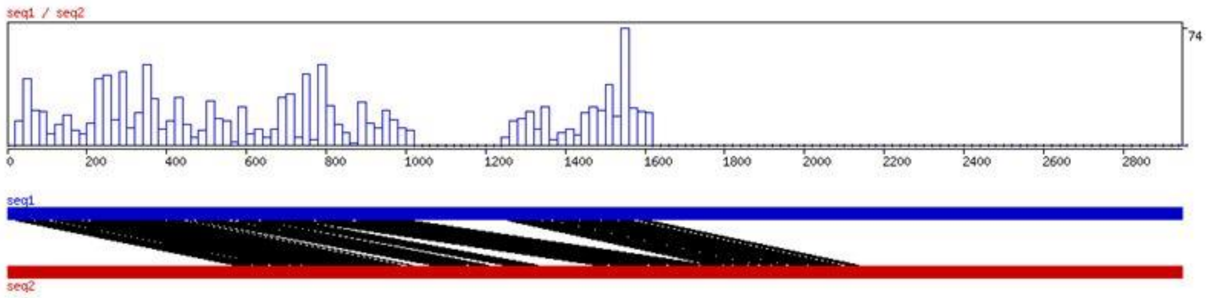

LAGAN

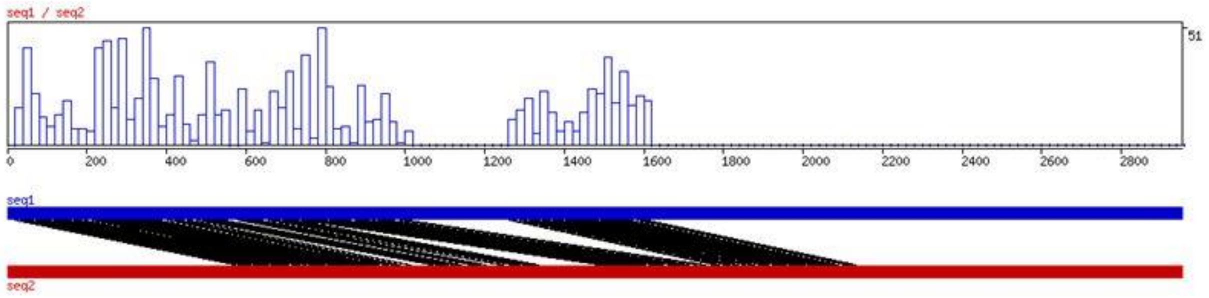

Figure S3. Seq 1: Ovis aries; Seq 2: Sus scrofa. Comparative sequence analysis of the sheep (*Ovis aries*) and pig (*Sus scrofa*) MC4R promoter regions (Accession nos. NM_001126370 and NM_214173, respectively) performed by CONREAL (**top**) and LAGAN methods (**bottom**). The graphs show the positions of aligned hits and the distribution/concentration of conserved TFBSs along the sequences. The graphs are followed by sequence-alignment data and tables of conserved TFBSs linked to TransFac entries (data not shown). Blue line above the black bar (sheep sequence) and the red line below the black bar (pig sequence) represent positions of the conserved regulatory elements in both species. The analysis parameters are 80% PWM threshold, 50% homology threshold, and 15-bp flank length.
